# The Institutional Library

**Published:** 1916-10-14

**Authors:** 


### THE INSTITUTIONAL LIBRARY.

**When to Advise Operation in General Practice.** By A. RENDLE SHORT, M.D., F.R.C.S. (Bristol: J. Wright and Sons. 1916. Pp. 280. Price 5s. net.)

THE medical profession already know—or ought to know—Dr. Rendle Short as a teacher who possesses originality of outlook, sound judgment, notable industry, and a capacity for getting to the root of any surgical question with a logical exposition. His already well-earned reputation for these by no means common merits and qualities will surely be enhanced by this eminently sane and readable monograph. It is wholly suitable to the purpose in view—namely, the weighing of the pros and cons as to operative or other treatment in all those cases where a conscientious practitioner is bound to put such a question in his own mind. To senior men it has the inestimable value of summing up judicially those advances in recent surgical method and technique which have invalidated much of the surgical teaching current twenty or thirty years ago; for those of junior standing it supplies the element of experience which no one not doing special surgical work can acquire except after many years of general practice. The author does not aim at finality: just as modern research has upset many of the conclusions which were absolutely legitimate a few years ago, so in turn he recognises that many of his teachings may in turn be upset in the future. Nor must it be expected that everyone will agree with him on every point; there are many topics dealt with in this book upon which reputable surgeons still differ. The present reviewer has been struck far more by the almost uniform assent he can give to Dr. Short's teachings than by the very far between and few occasions on which he feels compelled to differ. A physician might possibly cavil at the dismissal of typhoid fever (in a list of differential diagnosis) with the remark that the Widal reaction does not help in early cases, on the grounds that it is just in these early cases that blood cultivation is so likely to clinch the diagnosis and also that a quantitative daily Widal can give most valuable information even in fairly early cases. But the whole volume is so excellently conceived and so full of common-sense and sound teaching that criticism of any sort is almost ungracious. Dr. Short is not by any means of the type of surgeon to whom anything but instant operation for everything is foolishness. If anything, he inclines slightly towards conservatism, though he holds the scales between that and operative riotousness with great fairness and impartiality. The profession owes Dr. Short at the very least the compliment of procuring and studying his work without delay; there are few recent medical books so very well worth the price.

**Gray's Anatomy, Descriptive and Applied.** Nineteenth Edition. Edited by ROBERT HOWDEN, M.A., D.Sc. (London: Longmans and Co. Pp. 1,304. Price 32s. net.)

WARS and other ephemeral upheavals and catastrophes cannot, apparently, disturb the majestic progress from edition to edition of the premier text-book on anatomy in the English language. So much is "Gray's Anatomy" a part of the established order of things scientific that it is no longer thought necessary to prefix to it the prefaces written at various dates by its author and by others who from time to time have stepped into his shoes. Yet this ancient text-book is by no means fossilised by the passage of the years: if it were, it would soon cease to exist. It is so regularly and carefully revised and brought up to date that it maintains, and deserves to maintain, the popularity it has always enjoyed. Much of the old *pabulum* is there still upon which the studies of successive generations of medical students have been nourished; but there is much also that is new and different, as anyone whose medical education took place twenty or thirty years ago can see at a glance. Perhaps the most striking thing to such a glance is the contrast between the conservatism of surgeons and the more plastic minds of anatomists, as shown by the refusal of the former to adopt the international nomenclature which Professor Howden now uses so much as a matter of course that he does not think of referring to it. Perhaps the coming generation of surgeons, brought up upon "Gray," will do differently. Lastly, one must not omit to mention the excellence of the index, so often scamped in otherwise leading text-books.

**The Treatment of Diseases of the Skin.** By W. KNOWSLEY SIBLEY, M.D. Second Edition. (London: Edward Arnold. 1916. Price 6s. net.)

IT used to be said in connection with text-books on dermatology, as well as with regard to those on other specialities, that the sections devoted to treatment were their weakest part. The present volume, however, which has already reached its second edition, is wholly concerned with the various methods of therapeutics employed in diseases of the skin. The author is a great believer in the efficacy of $x$-rays, ionisation, and radium for many cutaneous disorders, and, accordingly, considerable space is occupied with a description of these agencies, whilst other physical methods, such as the carbonic-acid snow, high-frequency currents, Bier's hyperæmia, and electrolysis also find their place. It may be noted that the treatment of tuberculous affections of the skin by means of tuberculin is not given much prominence, in spite of the successes obtained by some authorities. The personal equation counts for a good deal with most clinical workers, and one is always inclined to lack enthusiasm regarding any therapeutic method which has not yielded the brilliant result in one's own hands that is said to have been achieved by others. It is with some surprise that we find the author stating that thyroid extract has "dropped out of favour of late" in the treatment of psoriasis. Many dermatologists still, we understand, believe this remedy to be of particular value in stubborn cases of psoriasis which have resisted all other means of treatment. A new remedy, in the shape of emetine hydrochloride in half-grain doses, is advocated by the author, in whose hands it has met with success in the treatment of psoriasis. The treatment of syphilis is fully discussed in close upon nineteen pages. It would have added more to the practical value of this chapter if concise directions had been given as to the actual methods of treatment, including the technique of intravenous medication, in a case of syphilis as it might present itself in private practice or in a hospital out-patient department. A useful list of 150 prescriptions is given, showing the class of case for which each may be employed. In two or three instances it appears to have slipped the mind of the author that certain preparations of German origin, obtainable before the war, are now no longer available, and, therefore, are inadmissible in prescriptions. On the whole, we consider that this text-book fully deserves the high place already accorded to it, and it may be strongly recommended to all practitioners and senior students.

**Mental Nursing.** By W. H. B. STODDART, M.D., F.R.C.P. Pp. 98. (London: The Scientific Press, 28 and 29 Southampton Street, W.C. Price 2s. 6d.)

DR. STODDART thinks that the would-be mental nurse should begin her work in an asylum, and after finishing her course there should proceed to a general hospital, and therein pass through a course of study in ordinary nursing, so that should she wish to devote herself to private nursing she would find herself doubly qualified for any post likely to offer itself. This is true enough; but we feel tolerably certain that the majority who have qualified in mental nursing will not readily exchange the liveliness and order of an asylum, and the hope of a pension, for any other kind of nursing work. So much the better for the asylum. One of the best and most useful chapters is that on epilepsy. So concisely and so clearly are the various phases of this disease given that it would be a most useful chapter for the general medical practitioner as well as the nurse to study. We hesitate to accept the view that epileptic patients may sometimes use their religious tendencies as a cloak for inhuman conduct. We believe them to be in the vast majority of cases perfectly sincere. Was it not epilepsy that at least contributed to the success of Mahomet? And should not Swedenborg be placed in the same class? Further on Dr. Stoddart tells the nurse that "Bibles, Prayer-books, and hymn-books have a detrimental influence on many patients, and their distribution requires the strictest supervision." This is indisputable; and in no class of the insane is it so often true as in the epileptic. The remarks on suicidal patients are so well put that they could hardly be improved on, and they should be carefully learnt by everyone likely to have charge of such cases. When an attempt at suicide is made the usual excuse of the nurse is, "I only left her for a minute"; but that minute may be fatal to the patient and most serious for the future career of the nurse. We feel sure that ere long a second edition will be called for, and, if so, Dr. Stoddart might correct the line from Goldsmith, unless, indeed, he intended the verbal inaccuracy to stand as more clearly descriptive of the "hollow" laugh of the dement.

**Uncensored Letters from the Dardanelles.** By A FRENCH MEDICAL OFFICER. (London: Heinemann. Price 3s. 6d.)

"LETTERS from the Dardanelles" is the proper title for this book. As they stand there is nothing in them to come within the province of any censor; and if there were, it is obvious that the censor would have deleted it so long as the war continues. Its real interest to English readers lies in the fact that its author is a Frenchman, attached as a medical officer to the Corps Expéditionnaire d'Orient, who somehow found time to write almost daily to his wife (an Englishwoman) from Alexandria, from Koum Kaleh, even Sedd-el-Bahr, and later Salonika, Strumnitza, and Topsin. His English wife hastened to translate (and print) the letters, some of which apparently appeared with his numerous snapshots in the French newspapers. A pleasant sense of France pervades these communications, which read like a diary, and have the point and verisimilitude of things seen and described without gloss or an eye on the public. The daily experiences of a doctor attached to the French forces in the Dardanelles are given with a certain point and even abruptness of description that makes agreeable reading, but there are no generalisations, and we notice as we read how concentrated and narrow a view of events is possible to those actively engaged in military operations. This limitation gives the work a certain vividness, and any document which preserves something of the atmosphere in which the great assault on the Dardanelles was carried out is worth reading to those who only remember the failure, and wonder how it struck an individual engaged in it. The nature of a retreat comes home to one as one reads the concluding description of the French retirement from Strumnitza to Salonika, and the final chapters form a bitter contrast to the previous gay life of Alexandria, which is described with much zest by the French doctor, who was always healthily alive to the beauty of nature and of women, and the varied appealing associations evoked by the site of Troy, the strange frescoes at Vatiluk, or the ladies' dresses at Alexandria. The medical organisation is touched on as lightly as any of the other conditions of life described. The author writes to his wife as a man of the world, not as a medical officer.

**Our Baby.** For Mothers and Nurses. By Mrs. J. LANGTON HEWER. Fifteenth Edition. Illustrated. (Bristol: John Wright and Sons, Ltd. 1s. 6d.)

THIS book is a first-rate guide for an intelligent mother who can provide her baby with all it needs without unnecessary luxury. The directions for the baby's toilet and clothing are excellent, and we are glad to note two points which modern nurses in their zeal for cleanliness are apt to overlook—namely, the exceeding delicacy of the new-born baby's skin. Therefore the writer advises that though "the mouth may be swabbed morning and evening with absorbent wool dipped in boracic lotion, the finger should not be used, as it is too hard and frequently causes an abrasion of the delicate mucous membrane, through which germs may enter, and thrush may develop. Many doctors now prefer that it be left untouched." Three-hourly intervals as a basis for both natural and artificial feeding are recommended, and the causes for failure in the former are admirably dealt with; while a whole section is devoted to the education of the child's digestive powers to bridge over the weaning period with as little disturbance as possible, and specimen diets up to two years are given. The chapter on artificial feeding begins with a warning against the pernicious practice of dosing with castor oil during the first few days, thereby robbing the bowel of its "antiseptic varnish," as the author happily describes the meconium. A wet-nurse is recommended in certain cases, to be obtained by inquiry amongst trained district midwives and examined by a doctor before engaging; and it is not considered necessary for the wet-nurse to wean her own baby, as a healthy woman can usually nurse two or even more. The methods of sterilising and pasteurising are clearly and concisely explained, and tables and receipts are given for whole and for modified milk. The directions for the care of premature and weakly babies, and the chapter entitled "Why Baby Does Not Get On," should be read by every mother and nurse, and will be found to solve many puzzles, while the directions for the moral training of infants are admirable.

**First Principles in Therapeutics.** By G. F. GOLDSBROUGH, M.D. (London: J. Bale, Sons and Danielsson. 1916. Pp. 138. Price 7s. 6d.)

FOR muddled and inchoate thought, for processes of obscure and incoherent reasoning based upon incomprehensible premisses, and for the long-drawn verbosity which conceals whatever meaning the author wishes to express, this expensive production can seldom have been surpassed in medical literature.

